# Deep Learning for Detecting COVID-19 Using Medical Images

**DOI:** 10.3390/bioengineering10010019

**Published:** 2022-12-22

**Authors:** Jia Liu, Jing Qi, Wei Chen, Yi Wu, Yongjian Nian

**Affiliations:** 1 Department of Digital Medicine, School of Biomedical Engineering and Imaging Medicine, Army Medical University (Third Military Medical University), Chongqing 400038, China; 2 Department of Radiology, Southwest Hospital, Army Medical University (Third Military Medical University), Chongqing 400038, China

The global spread of COVID-19 (also known as SARS-CoV-2) is a major international public health crisis. The epidemic has posed a threat to governments and societies worldwide, resulting in huge loss of life and property. The best way to stop the epidemic is to identify and isolate infected people as quickly as possible. Although many COVID-19 biomarkers and advanced sensing technologies have been proposed [1], reverse transcription polymerase chain reaction (RT-PCR) is the gold standard for COVID-19 detection [2]. Note that RT-PCR also has obvious disadvantages, such as false negative and poor real-time performance. In clinical practice, medical imaging is an effective tool for rapid auxiliary screening of COVID-19, such as CXR (Chest X-ray Radiograph) and CT (Computed Tomography). It was especially useful in the early stages of COVID-19 outbreak. In general, COVID-19 has certain features that differ from the other type of pneumonia in chest imaging; however, manual screening of COVID-19 from medical images is a time-consuming and labor-intensive task. In addition, it is difficult for clinicians with less experience to distinguish the imaging manifestations of COVID-19. Compared with the visual observation of clinicians, deep learning can automatically extract more separable features from chest imaging, greatly improve the diagnostic accuracy, and reduce the workload of clinicians. Therefore, scholars worldwide attempt to employ deep-learning techniques to handle medical imaging in order to achieve accurate COVID-19 detection.

As a kind of medical image that is easily obtained, CXR has been used to detect COVID-19. Minaee [3] carried out binary classification tasks to detect COVID-19 using four classical convolutional networks named ResNet18, ResNet50, SqueezeNet and DenseNet-121, and achieved the highest classification accuracy of 92.3%, which demonstrated the feasibility of COVID-19 detection using CXRs. Al-Waisy [4] tried to use two networks for decision fusion to reduce model bias, which carried out normal and abnormal classification tasks, and obtained a classification accuracy of 98.8%. This work initially explored the improvement space of COVID-19 intelligent detection tasks; however, this simple task is of little significance in practical application; it is inappropriate to classify normal and other diseases into the same category. Wang [5] employed some basic deep networks to detect normal chest, COVID-19 and other pneumonia, in which the sensitivity of COVD-19 can reach more than 80%. In addition, Wang released a large public dataset named COVIDx, which made great contributions to early research on COVID-19 detection. In addition, there are also some research studies on COVID-19 detection based on feature extraction. Features such as texture were extracted manually, and then classified by a neural network. Note that this operation increases workload, and the results are no better than those obtained using a neural network directly [6,7]. Lin [8] proposed an adaptive attention network (AANet) based on a deformable convolution network and self-attention mechanism, where adaptive deformable convolution could solve the problem of variable manifestations of COVID-19 lesions, and a self-attention mechanism could focus on the contextual information to handle lesions with complex manifestations in CXRs. In addition to traditional networks, some frontier deep networks have also been introduced. Park [9] used the CheXpert dataset to train a CNN (Convolutional Neural Network), where the extracted features from the input CXRs were used to train a Transformer to detect COVID-19. 

To solve the problem of limited training data, Oh [10] segmented the input CXRs into a number of patches. The trained network can classify each patch separately and vote for the final detection. It should be noted that in Oh’s work, bacterial pneumonia was considered as a separate category, while COVID-19 was combined with other viral pneumonia, which is disadvantageous for rapid screening of COVID-19 [11]. Zhang [12] employed the anomaly detection mechanism to make the training process focus on the class that needs to be detected, so as to enhance the detection performance of COVID-19. 

To improve the generalization capability, Liu [13] proposed the auxiliary learning strategy combined with implicit differential optimization to improve the generalization of the model, and the accuracy of COVID-19 detection reached 99.1%. Zhang [14] used neural dynamic learning and a bagging ensemble strategy to enable the network to dynamically adjust its own parameters for different samples in the testing phase, which obtained better results than the existing models used to detect COVID-19 and other viral pneumonia.

Compared with CXR, CT can provide clearer lung imaging, which is more helpful for the observation of pneumonia. Note that COVID-19 detection based on chest CT includes a two-dimensional (2D) approach and a three-dimensional (3D) approach, where the 2D approach is performed on each slice separately by the 2D deep network, and then a final decision can be made based on the detection results of all slices. A 3D approach is directly performed on the CT image to achieve a detection result. To improve the detection performance of deep learning, it is necessary to segment the pneumonia region on each slice in advance; however, it is very difficult to segment the pneumonia area due to the huge workload. A compromise can be reached by segmenting the double lungs on each slice, which can be conducted automatically by using deep learning.

There is relatively little literature concerning the 2D approach. Pathak [15] employed a deep transfer-learning technique to detect COVID-19-infected patients, where a top-two smooth loss function with cost-sensitive attributes was utilized to handle noisy and imbalanced COVID-19 dataset kind of problems. To enhance the generalization performance of the trained model, Wang [16] proposed a novel joint learning framework to detect COVID-19 by effectively learning from heterogeneous datasets with distribution discrepancy. Additionally, Qian [17] proposed a novel system that contains slice-level and patient-level classification networks to handle the discriminative feature learning from the spatial and temporal domain, respectively.

It is necessary to design an advanced attention mechanism to direct the focus of the convolutional network to the pneumonia area. This typically includes parallel attention [18], online attention [19], prior-attention residual learning [20] and multi-instance learning with dual attention [21]. 

Due to the limitations of the public CT dataset with ground truth label, weak supervised learning for 3D approach has been an important research field [22,23,24], which can achieve good detection performance with limited labeled samples. Even if the double lungs were not segmented in advance, the proposed algorithm achieved high performance of COVID-19 detection and lesion location [24]. In fact, a large-scale CT dataset with accurate annotation is still crucial for training a high-performance deep model; the release of relevant datasets can greatly promote the research process [25].

It can be seen that relevant research mainly focuses on the detection of COVID-19. In fact, in addition to COVID-19, there are many types of pathogens that cause lung infection, such as bacteria, viruses, fungi, mycoplasma, Chlamydia, etc. At present, it is still difficult to detect the above types of pneumonia clinically with high accuracy. Accurate detection of various types of pneumonia can better guide clinical treatment and improve prognosis. It can be predicted that the detection of multiple pneumonia will be a more challenging task than the simple detection of COVID-19. It should be noted that, for a patient with pulmonary pathogen infection, multiple pathogens may be infected at the same time, which undoubtedly increases the difficulty of intelligent detection of multiple pneumonia. In addition, early warning of pneumonia caused by new pathogens using deep learning is also a very meaningful task.

COVID-19 has been prevalent around the world for about three years, and most patients are asymptomatic, without obvious lung disease. Therefore, there has been relatively little detection of COVID-19 based on intelligent analysis of medical images. It should be noted that no matter how high the accuracy of COVID-19 detection based on a medical image is, it cannot replace RT-PCR. At present, clinical diagnosis based on artificial intelligence using medical images has become a research focus, which will play an important auxiliary function in future clinical diagnosis. Medical images usually contain rich information about lesions which are difficult to accurately and comprehensively identify. Intelligent analysis of medical images can provide fast and accurate disease detection, improve prognosis and significantly reduce clinical workload.

## Data Availability

Not applicable.
